# Does Intrauterine Exposure of the Foetus to Immunosuppressive Drugs Used by the Mother—The Organ Recipient—Affect the Development of Post-Vaccination Immunity against Selected Viral Diseases in Children of These Mothers in Postnatal Life?

**DOI:** 10.3390/vaccines11040738

**Published:** 2023-03-27

**Authors:** Tomasz Ginda, Karol Taradaj, Anna Stelmaszczyk-Emmel, Olga Tronina, Patrycja Kociołek, Oliver Jendro, Bożena Kociszewska-Najman

**Affiliations:** 1 Department of Neonatology and Rare Diseases, Faculty of Health Sciences, Medical University of Warsaw, 02-091 Warsaw, Poland; 2 Department of Laboratory Diagnostics and Clinical Immunology of Developmental Age, Faculty of Medicine, Medical University of Warsaw, 02-091 Warsaw, Poland; 3 Department of Transplantation Medicine, Nephrology and Internal Diseases, Faculty of Medicine, Medical University of Warsaw, 02-091 Warsaw, Poland

**Keywords:** immunogenicity, immunosuppressive drugs in pregnancy, transplantation, safety of vaccination, anti-viral vaccination, children

## Abstract

Background: Pregnancy in women who are organ recipients has long been a controversial issue due to the lack of data on the safety of immunosuppressive drugs for the developing foetus. Scientific data show that the effect of immunosuppressants on the foetus causes an impairment of T and B lymphocyte function and a reduction in their total number. For this reason, some authors recommend delaying the obligatory immunization of infants. The aim of the study is to analyse the impact of chronic immunosuppressive therapy used during pregnancy by women after organ transplantation on the effectiveness of anti-viral vaccinations in the children of these women. Methods: Concentrations of post-vaccination IgG antibodies (measles, HBV, polio) in 18 children of post-transplant mothers (9KTRs; 9LTRs) were determined using the ELISA method. The results were compared with the control group (*n* = 21). The incidence of vaccination AEs was also analysed. Results: There were no significant differences between the analysed groups in the concentrations of antibodies against HBV, measles and polio (*p* > 0.05). Conclusions: No difference was observed in the immunogenicity of HBV, polio and measles vaccinations between children of post-transplant mothers and the general population. The immunization of children of post-transplant mothers is safe, and the percentage of adverse post-vaccination events does not differ from the general population. The obtained study results do not indicate the necessity for modifying the vaccination program for HBV, measles, and polio in this group of patients.

## 1. Introduction

Transplantology is one of the most intensively developing fields of medicine. The constantly growing number of transplantations creates additional tasks during the period of broadly understood post-transplantation care. Both in the case of liver and kidney transplantation, organ recipients comprise patients in various age groups, including people of childbearing age. For a long time, pregnancy in female transplant recipients was a controversial issue due to the lack of data on the safety of immunosuppressive drugs for the developing foetus [1,2,3].

The first birth of a woman after an organ transplant took place in 1958. The patient’s pregnancy occurred after the transplant of a kidney, which the recipient received from her twin sister [4]. With the development of transplantology, immunology, perinatology, neonatology and other fields of medicine, pregnancy after organ transplantation became more common over the years, allowing women to enjoy the motherhood they always wanted [5,6,7].

Immunosuppressive drugs used after organ transplantation are known to have numerous side effects. Some are contraindicated for use in pregnant women due to the proven harmful effects on the developing foetus or even the teratogenic effects. All of these issues are significant in this group of patients, and therefore, it is extremely important to carry out a comprehensive analysis of the state of health and try to detect early on the risks that may occur in the children of post-transplant mothers not only for the mothers of these children and their families, but above all for the fates of the children themselves. In a group of newborns of post-transplant mothers, prematurity, low birth weight, or delivery of a wasted foetus are most frequently observed. When compared to newborns in the general population, these findings are confirmed by the literature.

Researchers focus on the multifactorial and long-term follow-up of children of post-transplant mothers. Several registries have been established worldwide to collect clinical data on these children’s development and potential health problems. The Transplant Pregnancy Registry International, which is the largest international database of publications on the course of pregnancies in women after organ transplantation and the postnatal life of their children, contains data on over 3000 pregnancies of women after organ transplantation. The registry is based on the principles of Evidence-Based Medicine. The aim of the registry is to provide comprehensive recommendations on aspects of pregnancy management to recipients and to provide guidelines for the postnatal care of their children [8]. Despite the presence of important clinical issues in the care of children of post-transplant mothers, the topic of preventive vaccination has not been discussed so far, and it remains open to researchers.

Vaccines are a cornerstone in the long and challenging construction of better global health. Neonatologists and paediatricians who take care of children of post-transplant mothers often wonder whether—due to the higher percentage of prematurity, obstetric complications, and impaired development of leukocytes in the first months of life [4]—a different (deferred) vaccination program should be used. The issue of preventive vaccinations, including specific recommendations regarding vaccinations of children of mothers that are recipients, has not been widely discussed so far. Therefore, there are no unequivocal recommendations from specialists regarding the course of vaccination in this particular group of patients.

Taking into account the fact that obligatory vaccination schedules vary from country to country, the vaccination schedule in Poland, which was implemented equally by all study participants, is presented below.

Hepatitis B Virus vaccine: the first dose up to 24 h after birth, the second dose 3 months after birth, the third dose 7 months after birth. Polio vaccine: the first dose (IPV—Inactivated Poliovirus Vaccine) 3–4 months after birth, the second dose (IPV) 5–6 months after birth, the third dose (IPV) 16–18 months after birth, and the fourth dose (OPV—Oral Poliovirus Vaccine) at the age of 6. Measles vaccine (MMR—Measles, Mumps, Rubella vaccine): the first dose 13–14 months after birth, the second dose at the age of 10.

The aim of this study is to compare the immunogenicity of vaccination against selected childhood infectious diseases of viral aetiology (hepatitis B, polio, measles) based on the assessment of post-vaccination IgG antibodies from long-term follow-up in children of post-transplant mothers and children not exposed to immunosuppressive drugs in the prenatal period (from the general population).

In addition, the aim is to determine whether the type of transplanted organ (liver or kidney) from a mother and the subsequent immunosuppressive treatment regimen used prenatally influences the immunogenicity of vaccination in the child.

## 2. Materials and Methods

The study group consisted of 18 children aged 6–16 (born in 2008–2014) whose mothers during pregnancy took immunosuppressive drugs due to liver or kidney transplantation. The most commonly used immunosuppression regimen in renal recipients was cyclosporine + azathioprine + steroid, and in the liver recipient group, tacrolimus + azathioprine + steroid.

The control group consisted of 21 children recruited from the general paediatric population who were without exposure to immunosuppressive treatment in the prenatal period. The children were matched to the study group in terms of age. Three of the children in the control group were under 10 years of age (2 girls aged 6 years, 1 boy aged 7 years). Thus, the size of the control group for measles was correspondingly smaller and consisted of 18 children. They received only one dose of MMR vaccination (a booster dose is given at 10 years of age). These children were not included in the statistical analysis for measles. They had the same doses of the other vaccines (HBV, polio) as the rest of the children.

The inclusion and exclusion criteria are presented in Table 1. A detailed description of the groups is shown in Table 2. The study was conducted in accordance with the principles of the Declaration of Helsinki, and the study protocol was approved by the Medical University of Warsaw Bioethics Committee (Approval no. KB/161/2021).

All children were vaccinated against hepatitis B, polio, and measles on schedule, in accordance with the immunization program applicable for their birth year.

A measure of 3 mL of venous blood was collected from each patient into a clot tube. The material was centrifuged 1.5 h after being collected in a centrifuge (10,000 rpm, 5 min). Then, the material was frozen at −80 degrees Celsius. The material had been stored until ELISA tests were performed.

ELISA tests were performed using standardized kits by Alpha Diagnostic Intl., Inc. San Antonio, Texas, USA. The tests for each sample were performed twice. The tests were performed in accordance with the manufacturer’s instructions [9,10,11].

Absorbance readings were performed using a UVM340 plate reader (ASYS, Biogenet), and the results were analysed using MikroWin2000 v4 software (Mikrotek Laborsysteme GmbH, Biogenet, Overath, Germany). Antibody concentrations are expressed in conventional units (U/mL), which are defined by the manufacturers for each test as appropriate. The details are presented in Table 3.

A survey was conducted on the children’s general health, past illnesses, hospitalizations, and adverse post-vaccination events. The questions that were included in the form along with the results are presented in Table 2. Data on vaccination adverse events were obtained through a questionnaire for the parents of the children.

### Statistical Analysis

Mean concentrations of post-vaccination IgG antibodies against the following viruses were analysed: hepatitis B, measles and polio. Mean antibody concentrations were the arithmetic mean of antibodies calculated from two independent absorbance readings for individual patient sera.

For the parameters analysed (separately for the control and study groups and the subgroups of the study group: LTRs and KTRs), the conditions for parametric tests were verified. The normality of distribution was assessed using the Shapiro–Wilk test. The homogeneity of variance was tested using the Levene’s test.

For each of the three analysed tests (polio, hepatitis B, measles), the data did not meet the assumptions for parametric tests (the null hypothesis of normal distributions had to be rejected, *p* < 0.05).

In view of the above, the results were subjected to non-parametric analysis. Two separate statistical analyses were performed. The significance of differences in antibody titres between the control and study groups was analysed using the Kolmogorov–Smirnov test for two independent samples. The ANOVA signed rank Kruskal–Wallis test was used to investigate the significance of differences in antibody titres between the groups, taking into account the type of mother’s organ transplantation. The Kolmogorov–Smirnov test is based on the maximum absolute difference between the observed cumulative distribution functions for both samples. Kruskal’s test is the most powerful non-parametric equivalent to the parametric analysis of variance. The choice of the tests was attributed to the small size of the control and study groups, which resulted from the unique nature of the group of patients, which included children of post-transplant mothers.

## 3. Results

**Figure 1:**
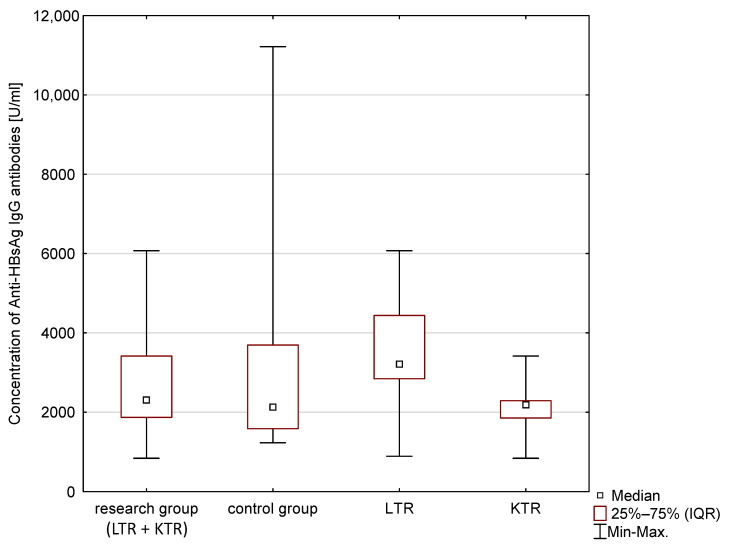
Concentration of Anti-HBsAg IgG antibodies in blood serum.

**Figure 2:**
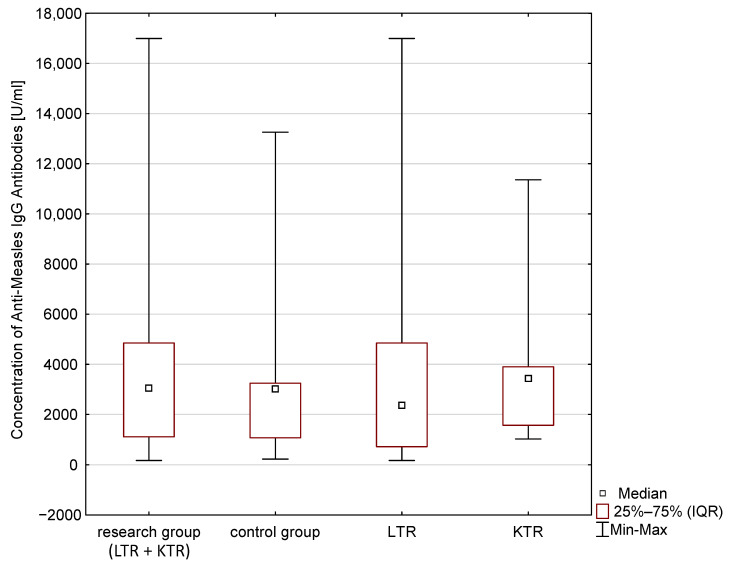
Concentration of Anti-Measles IgG antibodies in blood serum.

**Figure 3:**
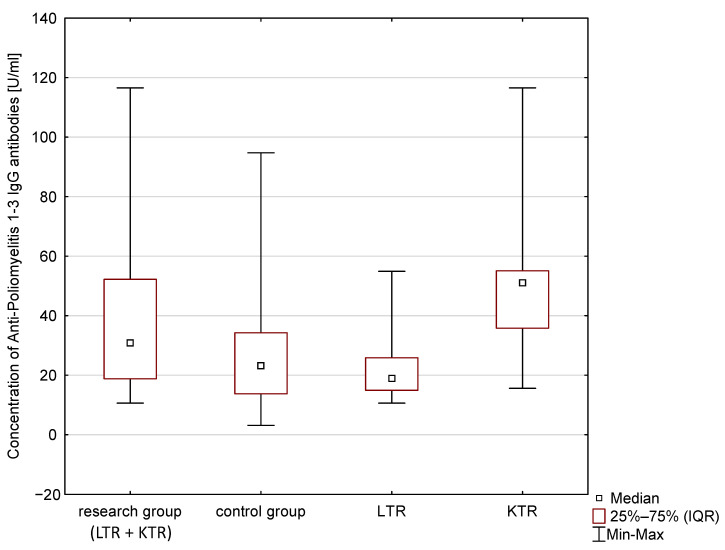
Concentration of Anti-Poliomyelitis Virus 1-3 IgG antibodies in blood serum.

The statistical analysis results are presented in Table 4 and Figure 1, Figure 2 and Figure 3. The median concentration of anti-HBV antibodies (Anti-HBsAg), anti-poliovirus antibodies and anti-Measles antibodies are presented. These results, however, are not statistically significant (*p* > 0.05). The Anti-Poliomyelitis virus 1–3 IgG and anti-measles virus antibodies (Anti-Measles IgG) in the group of children of post-transplant mothers was higher compared to the control group. The differences expressed by the Kolmogorov–Smirnov test were not statistically significant (*p* > 0.1). The obtained concentrations of post-vaccination immune antibodies (Anti-Poliomyelitis virus 1–3 IgG, Anti-HBsAg IgG, Anti-Measles IgG) were interpreted according to the recommendations of the manufacturer of the ELISA tests (details in Table 3).

As presented in Table 5, all children obtained protective levels of post-vaccination antibodies against HBV and measles. The exception is the Anti-Poliomyelitis virus 1–3 IgG antibodies, in the case of which nearly 10% of the cases from the general paediatric population were seronegative after vaccination. The result is interesting because it only applies to the general population. However, these results are not statistically significant (*p* > 0.05).

The ANOVA signed rank Kruskal–Wallis test was used to analyse the concentrations of immune antibodies in children of mother recipients. It took into account the type of transplantation. The detailed results are presented in Table 6. No statistically significant differences were found, despite the differences in the median concentrations of the tested antibodies between the groups (*p* > 0.05).

## 4. Discussion

The results of our study showed no statistically significant differences in the concentrations of post-vaccination antibodies against the HBV virus, measles virus, and poliovirus between children of mothers who were liver and kidney recipients and those from the general population. It is very tough to compare the results with other researchers due to the limited number of publications on the health of children of mothers who had received liver and kidney transplantation.

Due to the relatively rare occurrence of HBV, polio, and measles in modern Europe, the contribution of patient contact with these viruses to immunity is insignificant. Consequently, the results obtained primarily illustrate the effect of the vaccine response.

As mentioned in the introduction, there are only a few centres that keep records of the development of children of post-transplant mothers. Follow-ups in our centre are carried out from the moment a child is born to an organ recipient to when they reach the age of majority, and sometimes also after reaching the age of majority. The long-term specialist care of children (0–18 years) by our team means that the follow-up data on the health problems of the patients is constantly recorded, and it becomes possible to detect clinically interesting differences in the development of this group of children in relation to the general population.

An extensive analysis of the worldwide literature published in the last thirty years was carried out (1992–2022) during the search for research on the development and function of the immune system in children of post-transplant mothers. This research included the comparison of the titres of post-vaccination antibodies against viruses such as HBV, measles, and polio in the indicated group with the general paediatric population.

One study was found that evaluated post-vaccination responses in children of mother-recipients that were immunized against bacterial diseases, but not viral diseases as was the case of our study. Dinelli et al. [12] examined humoral response after vaccination against Haemophilus influenzae, S. pneumoniae, and M. tuberculosis in 24 children of mother-recipients and found no differences in the immune response compared to the control group (*p* > 0.05). These results were consistent with our observations, suggesting an adequate immune response in children of post-transplant mothers to preventive vaccinations. However, this team’s research results cannot be directly compared with ours because both groups tested the response against different vaccines.

No article was found that compared post-vaccination antibody titres against HBV virus, measles virus, and poliovirus in the indicated group compared to the general paediatric population. Consequently, the results of this study are pioneering on a global scale. At the same time, due to the lack of studies available for comparison, it is not possible to relate the obtained results directly to the results of other researchers.

In the data bases of medical publications, a few articles were found whose authors examined the function and development of the immune system in newborns and infants of post-transplant mothers. Schen et al. [13], in a 12-month follow-up period, showed a reduced number of T lymphocytes (CD3+, CD4+, CD8+) and B lymphocytes (CD19+) in a white blood cell smear in 11 children of mothers who had undergone kidney transplantation compared to children of mothers who had not been exposed to immunosuppressive drugs in the prenatal period. Moreover, Schen et al. [13] found reduced levels of immunoglobulins in the same group of children. E. Ono et al. [14] examined 28 children after delivery and in the 8th month of life and showed a similar dependence on a lower number of lymphocytes to that of Schen et al. [13]. They also pointed out the necessity of the close monitoring of infants of mothers after organ transplantation who may be at higher risk of hospitalization not only due to prematurity, but also due to the fact they were born to female organ recipients. Due to the lack of studies on this issue in the literature, studies conducted on very small groups of patients, as in the case of Di Paolo et al. [15] and Takahashi et al. [16] (in both studies, groups of six newborns), who confirm this correlation, cannot be omitted. Based on the results, some researchers draw conclusions in which they recommend delaying mandatory vaccinations in this group of infants [4]. Some authors suggest that the first doses of vaccine should be administered after the 6th month of life and not after the 6th week of life [4], as in the case of the general population of children. They present the problem of both the possibility of a suboptimal immune response and the increased risk of vaccine adverse events.

The data presented above is most likely the result of immunosuppressive treatment used during pregnancy. Freriksen et al. [17] showed that tacrolimus, which is widely used in transplantation, may accumulate in the tissues of the foetal placenta. All immunosuppressive drugs used after kidney and liver transplantation cross the placental barrier and can be detected in foetal blood [17].

Taking into account our results, it seems that alterations to the white blood cell line of the immune system occurring in the first months of life do not affect the ability to develop long-term, specific post-vaccination immunity against viral diseases.

Our study did not record an increased percentage of adverse post-vaccination events compared to the general population for the analysed vaccinations. This analysis proves the safety of vaccinations that are carried out according to the standard program in children of mothers who undergo organ transplantation.

To date, no studies have been conducted that could provide the basis for creating recommendations regarding vaccinations in children of post-transplant mothers. Currently, immunization in this group is the same as that in the general population. There is no argument as to whether such a procedure is correct or involves an increased risk for children of mothers that are organ recipients. Taking into account our results, it seems reasonable to maintain a standard vaccination protocol in the group of children of post-transplant mothers that would be the same as for the general population. Due to the known mechanisms of transfer of immune antibodies through the placenta and breast milk, active screening of antibody levels in organ recipients who plan to become pregnant should be considered in order to possibly administer an additional dose of the vaccination. Such actions may provide better protection in the early postnatal period when children of post-transplant mothers have reduced lymphocyte counts.

The obtained conclusions require confirmation through studies conducted on more numerous groups of children of post-transplant mothers.

The results of studies of other clinical parameters in children of mothers after transplantation published in recent years mostly show no differences in the parameters studied when compared to children from the general paediatric population. Such findings were observed in papers in relation to lipid profile, biochemical parameters, and neurological development of these children [18,19,20].

These data indicate that there is a non-existent or insignificant impact of the immunosuppression used during pregnancy on the long-term development of their children. The number of pregnancies of post-transplant mothers is successively increasing, and these mothers can enjoy the motherhood that they longed for.

## 5. Limitations of the Study

This study analysed post-vaccination antibody titres against HBV, polio, and measles in subjects aged 6–16 years. This study does not allow us to determine whether the immunological response in the earlier years of life (under 6 years) is comparable. The study assessed only the humoral response. On its basis, it is impossible to determine the role of the cellular response in developing post-vaccination immunity against virus diseases. The objective of the study was to determine immunogenicity. Still, it is difficult to assess the real effectiveness of vaccination because of the low incidence of hepatitis B, poliomyelitis, and measles in Europe due to collective immunity caused by the high vaccination coverage of Europeans. Antibody titres are not the only measure of post-vaccination protection. The immune memory response as measured by an increase in antibody titres after a booster dose may be an equally important factor in protection against disease when compared to baseline titres alone. The statistical analysis of the effect of specific immunosuppressive drugs used during pregnancy on postvaccination antibody concentrations was not conducted. This was due to the large variation in immunosuppressive therapy regimens, which, with a small sample size, would have made it impossible to obtain objective results and thus statistical inference.

## 6. Conclusions

No difference was observed in the immunogenicity of hepatitis B, polio, and measles vaccinations between children of post-transplant mothers and those of the general population. Based on the study, there are no differences in the immunogenicity of vaccinations in the child and the type of organ transplanted to the mother.

The immunization of children of mothers that have had organ transplantation is safe, and the percentage of adverse post-vaccination events does not differ from the general population.

The obtained results do not indicate the need for modification of the vaccination program against HBV, measles, and polio in this group of patients.

## Figures and Tables

**Table 1:** Inclusion and exclusion criteria.

Inclusion Criteria	Exclusion Criteria
Age: 6–16 Maternal immunosuppression during pregnancy due to organ transplantation Non-use of long-term pharmacotherapy Immunization in accordance with the Protective Immunization Program applicable in Poland for this year of birth Informed consent to participate in the study	Active infection of the respiratory tract, digestive system, and urinary tract within 30 days prior to sample collection (existence of symptoms such as runny nose, cough, body temperature above 38 degrees Celsius, acute diarrhoea) Chronic diseases of the digestive system (e.g., Crohn’s disease), respiratory system (e.g., cystic fibrosis), in particular autoimmune diseases, systemic connective tissue diseases, congenital and acquired immunodeficiencies, cancer

**Table 2:** Detailed description of the study and control groups.

Parameters	Transplant n = 18	Control n = 21	*p* Value (Mann-Whitney U Test)
Children			
Male	7 (39%)	9 (43%)	*p* > 0.05
Female	11 (61%)	12 (57%)	*p* > 0.05
Mean ± SD age	12.11 ± 3.16	9.05 ± 3.07	*p* > 0.05
Chronic diseases	2 (11%)	3 (14%)	*p* > 0.05
History of hospitalization	0 (0%)	1 (5%)	*p* > 0.05
History of vaccination AEs			
Mild	3 (17%)	5 (24%)	*p* > 0.05
Moderate	0 (0%)	0 (0%)	*p* > 0.05
Severe	0 (0%)	0 (0%)	*p* > 0.05
Type of Tx			
Kidney	9		
Liver	9		
Immunosuppressive schemes during pregnancy	Children of KTRs	Children of LTRs	
Cyclosporine + azathioprine + steroid	6 (67%)	0	
Tacrolimus + azathioprine + steroid	3 (33%)	3 (33%)	
Tacrolimus + steroid	0	1 (11%)	
Tacrolimus	0	2 (22%)	
Azathioprine + steroid	0	1 (11%)	
Tacrolimus + azathioprine	0	2 (22%)	

KTRs—kidney transplant recipients, LTRs—liver transplant recipients, Tx—transplantation AEs—adverse events.

**Table 3:** Interpretation of the results of ELISA tests concerning Anti-Poliomyelitis virus 1–3 IgG, Anti-HBsAg IgG, and Anti-Measles IgG, according to manufacturers’ recommendations.

	Anti-Poliomyelitis Virus 1–3 IgG	Anti-HBsAg IgG	Anti-Measles IgG
Interpretation:	Positive > 10 U/mL Negative < 10 U/mL	Positive > 10 U/mL Negative < 10 U/mL	Positive 12 U/mL Not conclusive 8–12 U/mL Negative < 8 U/mL

**Table 4:** Median and IQR (*Interquartile range*) of post-vaccination antibody concentrations (Anti-Poliomyelitis virus 1–3 IgG, Anti-HBsAg IgG, Anti-Measles IgG) in children of post-transplant mothers and in children from the general population.

	Children of Post-Transplant Mothers		Children from the Control Group		Results of the Analysis Using the Kolmogorov–Smirnov Test		
	Median Concentration of Antibodies IgG [U/mL]	IQR [U/mL]	Median Concentration of Antibodies IgG [U/mL]	IQR [U/mL]	Maximum Negative Difference	Maximum Positive Difference	*p*
HBV	2306.04	1546.34	2130.93	2109.38	−0.14	+0.21	*p* > 0.1
Polio	30.84	33.41	23.21	20.48	0.00	+0.30	*p* > 0.1
Measles	3052.66	3736.53	3020.19	2173.48	−0.06	+0.26	*p* > 0.1

**Table 5:** Seropositivity after vaccination against hepatitis B, polio, and measles in children of post-transplant mothers (study group) and children from the general paediatric population (control group).

	Anti-HBsAg IgG		Anti-Measles IgG		Anti-Poliomyelitis Virus 1–3 IgG	
	Study Group	Control Group	Study Group	Control Group	Study Group	Control Group
Seropositivity	100%	100%	100%	100%	100%	90.5%

**Table 6:** Median and IQR of post-vaccination antibody concentrations (Anti-Poliomyelitis virus 1–3 IgG, Anti-HBsAg IgG, Anti-Measles IgG), taking into account the type of transplantation.

	Children of Mothers after Kidney Transplantation		Children of Mothers after Liver Transplantation		Control Group	
	Median Concentration of Antibodies IgG [U/mL]	IQR [U/mL]	Median Concentration of Antibodies IgG [U/mL]	IQR [U/mL]	Median Concentration of Antibodies IgG [U/mL]	IQR [U/mL]
HBV	2187.50 *	436.42	3211.21 *	1594.83	2130.93 *	2109.38
Polio	51.11 *	19.31	18.97 *	10.94	23.21 *	20.48
Measles	3440.09 *	2335.51	2362.43 *	4133.18	3020.19 *	2173.48

* differences between the individual groups were not statistically significant (*p* > 0.05).

## Data Availability

The data presented in this study are available on request from the corresponding author.
